# Food Restriction in Mice Induces Food-Anticipatory Activity and Circadian-Rhythm-Related Activity Changes

**DOI:** 10.3390/nu14245252

**Published:** 2022-12-09

**Authors:** Theo Gabloffsky, Sadaf Gill, Anna Staffeld, Ralf Salomon, Nicole Power Guerra, Sarah Joost, Alexander Hawlitschka, Markus Kipp, Linda Frintrop

**Affiliations:** 1 Institute of Applied Microelectronics and Computer Engineering, Faculty of Computer Science and Electrical Engineering, University of Rostock, 18119 Rostock, Germany; 2 Institute of Anatomy, Rostock University Medical Center, 18057 Rostock, Germany

**Keywords:** anorexia nervosa, activity-based anorexia, running wheel activity, food-anticipatory activity, circadian-rhythm-related activity

## Abstract

Anorexia nervosa (AN) is characterized by emaciation, hyperactivity, and amenorrhea. To what extent AN-related symptoms are due to food restriction or neuronal dysfunction is currently unknown. Thus, we investigated the relevance of food restriction on AN-related symptoms. Disrupted circadian rhythms are hypothesized to contribute to the pathophysiology of AN. Starvation was induced by restricting food access in early adolescent or adolescent mice to 40% of their baseline food intake until a 20% weight reduction was reached (acute starvation). To mimic chronic starvation, the reduced weight was maintained for a further 2 weeks. Locomotor activity was analyzed using running wheel sensors. The circadian-rhythm-related activity was measured using the tracking system Goblotrop. Amenorrhea was determined by histological examination of vaginal smears. All cohorts showed an increase in locomotor activity up to 4 h before food presentation (food-anticipatory activity, FAA). While amenorrhea was present in all groups except in early adolescent acutely starved mice, hyperactivity was exclusively found in chronically starved groups. Adolescent chronically starved mice showed a decrease in circadian-rhythm-related activity at night. Chronic starvation most closely mimics AN-related behavioral changes. It appears that the FAA is a direct consequence of starvation. The circadian activity changes might underlie the pathophysiology of AN.

## 1. Introduction

Anorexia nervosa (AN) is an eating disorder that is characterized by significant body weight loss and amenorrhea [1]. This psychiatric disorder is a common chronic disease during adolescence associated with a high mortality rate. Up to 80% of AN patients develop hyperactivity, which is commonly interpreted as a strategy to burn calories [2,3]. Excessive exercise is associated with a higher hospitalization period [4] and poor treatment outcomes [5] indicating hyperactivity as a severe problem in the starvation process. In addition, AN patients develop circadian and sleep–wake rhythm disturbances which might be involved in the underlying pathophysiology of eating disorders [6,7]. 

Translational animal research may help to unravel the pathophysiology and the relevance of severe food restriction on AN-related symptoms. For this purpose, the original activity-based anorexia (ABA) model in rats and mice, which combines food restriction (ad libitum food intake of 1–3 h per day) with running wheel access, mimics many of the somatic aspects of the illness, including body weight reduction, amenorrhea, and hyperactivity (reviewed in Refs. [8,9]). Paradoxically, the animals run voluntarily not only during the starvation period but also during feeding times. Under specific conditions, some animals run themselves to death [10]. To investigate chronic food restriction, we established a modified ABA model that avoids the high mortality rate observed in the original model, whilst including a fixed level of body weight loss maintained over a longer period [11,12]. In these animals, extensive running has been interpreted as food-seeking behavior [13]. In addition to this general hyperactivity, food restriction led to a so-called food anticipatory activity (FAA), an enhancement of running wheel activity before the feeding periods. There is evidence that in mammals, FAA might be regulated by a master pacemaker which is neuroanatomically localized in the suprachiasmatic nucleus (SCN) of the hypothalamus [14]. The SCN is responsible for synchronizing the circadian rhythm of daily light-dark phases. A change in this rhythm might be induced by food restriction in nocturnal animals [15,16]. Two studies already demonstrated that ABA animals changed this circadian activity, i.e., an increased activity during the light phase [17,18]. Whether circadian activity rhythm disruptions underlie the pathophysiology of AN is currently unknown.

In summary, the underlying pathophysiology of AN, and especially the causal relationship between AN-related symptoms such as hyperactivity and amenorrhea, on the one hand, and food restriction or neuronal dysfunction on the other hand, is mostly unknown. Therefore, we aim to investigate the contribution of circadian and sleep–wake rhythm disturbances to AN pathophysiology. The underlying processes might provide new insights into disease mechanisms and treatment options of AN which are very limited. Furthermore, in this study, we aim to determine whether acute and chronic starvation induces AN-related symptoms hypothesizing that the mice demonstrate amenorrhea and hyperactivity. It is unknown whether the intensity of FAA changes during the course of chronic starvation, potentially worsening the severity of the disease over time. Furthermore, we analyzed whether starvation changes circadian- and sleep–wake-rhythm-related activity, speculating that the mice decrease their activity during the night. For this purpose, we used an infrared-sensory-based tracking system (Goblotrop) that measures the time the mice spend in different parts of the cage.

## 2. Materials and Methods

### 2.1. Animals

Female C57BL/6J mice (*n* = 60; early adolescent—4 weeks old; and adolescent—8 weeks old) were purchased from Janvier Labs (Le Genest-Saint-Isle, France) and maintained under a 12/12 h light/dark cycle (lights on at 6 a.m.) and controlled temperature of 22 ± 2 °C. We focused this study on female mice given the higher prevalence of AN in female patients in comparison to male patients [19,20,21]. This also allowed us to use amenorrhea as an AN-related symptom representing an adequate level of starvation. The cages housing one mouse per cage were changed once per week and microbiological monitoring was performed according to the Federation of European Laboratory Animal Science Associations (FELASA) recommendations. All experimental procedures were approved by the Review Boards for the Care of Animal Subjects of the district government of Mecklenburg-Western Pomerania (reference number 7221.3-1-005/21). 

### 2.2. Study Design 

The induction of the ABA model was previously described [11]. In brief, after an acclimatization phase of 10 days with ad libitum access to food and water and daily animal handling, the mice were randomly assigned to treatment groups on the first experimental day. During the experiment, the body weight, food consumption (measured by daily weighing of the food), and menstrual cycles of the mice were recorded daily at 1 p.m. during the light phase. At this time point, food was provided to the mice. The extent of wheel running was measured using an exercise wheel (11.5 cm in diameter) mounted to the top of a standard mouse cage. The running distance was monitored once per hour with an activity software (VitalView Activity 1.4, STARR Life Science Corp., Oakmont, PA, USA). 

Two different models were used in this study, acute and chronic starvation. The acute starvation phase included one week of starvation in which the mice received 40% of their average daily food intake until a 20% body weight loss was achieved (Control_acute_4W: *n* = 5; ABA_acute_4W: *n* = 10; Control_acute_8W: *n* = 5; ABA_acute_8W: *n* = 10). When this body weight loss was reached, the food intake was adjusted daily to maintain the 20% weight loss.

**Figure 1:**
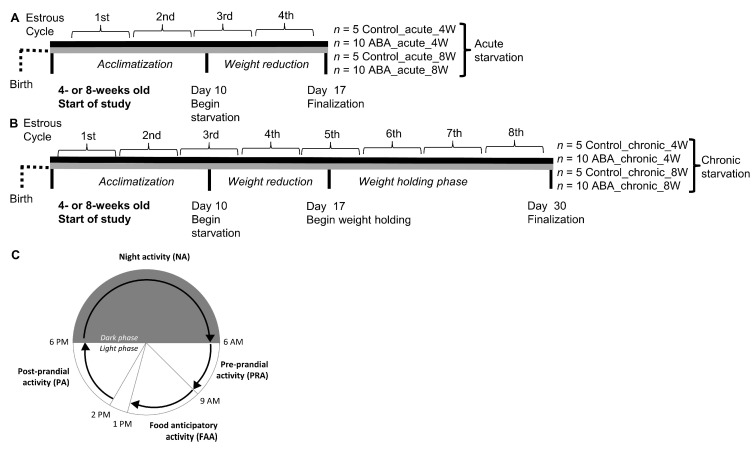
A schematic summary of the ABA model. (**A**) Schematic depicting the experimental study to induce acute starvation in (i) early adolescent (4 weeks old) and (ii) in adolescent (8 weeks old) mice. (**B**) Schematic depicting the experimental study to induce chronic starvation in (i) early adolescent and (ii) adolescent mice. Body weight, running wheel activity and estrous cycle were analyzed daily. The 4-day blocks of estrous cycle are presented in the chronological course of the model. (**C**) To measure the different daily periods of running activity, the experimental structure of cycles of 24 h with the following phases was used: food-anticipatory activity (FAA, 4 h, from 9 a.m. to 1 p.m.), post-prandial activity (PA, 4 h, from 2 p.m. to 6 p.m.), night activity (NA, 12 h, 6 p.m. to 6 a.m. next day) and pre-prandial activity (PRA, 3 h, from 6 a.m. to 9 a.m.).

To mimic chronic starvation, the acute starvation phase was followed by another two weeks of starvation (Control_chronic_4W: *n* = 5; ABA_chronic_4W: *n* = 10; Control_chronic_8W: *n* = 5; ABA_chronic_8W: *n* = 10). In this phase, the mice received 60–80% of their average daily food intake (of the acclimatization phase). When the body weight differed by more than 2.5% compared with the target weight, the food quantity was increased or decreased in increments of 5%. The control groups were housed under the same conditions but were fed ad libitum during the whole experiment. Figure 1 shows a schematic summary of the experimental setup. 

### 2.3. Locomotor Activity Determination

Each animal was housed individually in a cage with a running wheel purchased from STARR Life Science Corp. (Oakmont, PA 15139, USA). The running wheel activity was monitored with an activity software (VitalView Activity 1.4, STARR Life Science Corp.) via a digital magnetic counter which was attached to the wheel and connected to a microprocessor that registered the number of revolutions per hour. For analysis of the running wheel activity, different periods were defined (Figure 1C): FAA (4 h, from 9 a.m. to 1 p.m.), post-prandial activity (PA, 4 h, from 2 p.m. to 6 p.m.), night activity (NA, 12 h, 6 p.m. to 6 a.m. next day) and pre-prandial activity (PRA, 3 h, from 6 a.m. to 9 a.m.). The feeding time (1 p.m. to 2 p.m.) was excluded from the analysis. 

### 2.4. Determination of the Estrous Cycle 

The estrous cycle was determined as previously described [11]. Vaginal smears were taken, fixated in methanol, and stained with 10% Giemsa solution (Giemsa stock solution, ROTH T862.1, TH.GEYER, Hamburg, Germany). The cell morphology of the vaginal smears was analyzed under a microscope using 20- and 40-fold objectives to stage the estrous cycle in estrous, metestrous, diestrous, and proestrous phases. The estrous phase is characterized by the occurrence of cornification and clustering of epithelial cells, whereas the other phases are characterized by the presence of leukocytes and nucleated epithelial cells (Supplementary Figure S1). Amenorrhea was defined as no detectable fertile phase (estrous phase) within the regular duration of the murine cycle, id est, 4 days. Therefore, the percentage of mice with an estrous phase in 4-day blocks was calculated.

### 2.5. Goblotrop System

**Figure 2:**
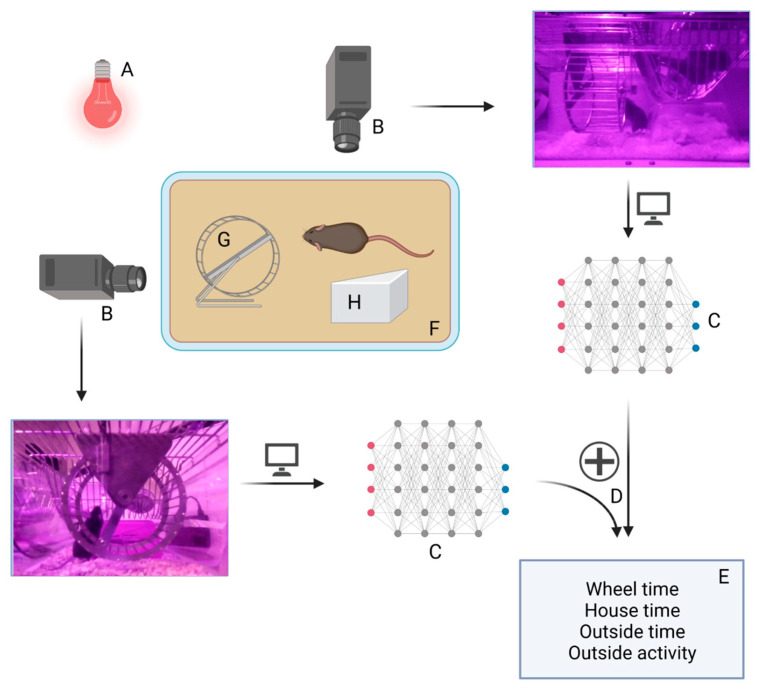
Overview of the different components of the Goblotrop system. Overview of the different components of the Goblotrop system. (**A**) infrared illumination, (**B**) infrared cameras from different perspectives, (**C**) neural networks, (**D**) fusing the output of the neural networks into parameters of interest, (**E**) parameter of interest, (**F**) cage of the mice, (**G**) running wheel, (**H**) house. Created with BioRender.com.

To determine the effect of starvation on circadian- and sleep–wake-rhythm-related activity, we used an infrared-illuminated deep artificial intelligence-based home-cage video tracking system called the Goblotrop. A sample of 8-week-old mice was subjected to the system for 22 h at the end of each of the three experimental phases, id est the phase of acclimatization, acute and chronic starvation phase (Control_chronic_8W: *n* = 4; ABA chronic_8W: *n* = 8). The Goblotrop consists of four recording stations, each consisting of two cameras (a front and side camera) and a neural network to determine the position of the mouse in each frame of the video (Figure 2). An evaluation software matched the position of the mice with defined areas within the cage. The following parameters were evaluated: (i) time spent inside the running wheel: wheel time, (ii) time spent in the house: house time, (iii) time spent outside of the house and the running wheel: outside time, and (iiii) the distance the mouse moved outside of the house and the running-wheel: outside activity. All measured parameters were summed up hourly. Each camera captured videos with a resolution of 1640 × 920 px (25 frames per second, FPS). The position of the mouse in the videos was detected by a neural network.

### 2.6. Statistics

Data are represented as means and standard errors of the mean (SEM). For statistical testing, the values for body weight and running wheel activity were compared for the acclimatization phase (days 1–10), the acute starvation phase (days 11–16) and the chronic starvation phase (days 17–29). The running activity was summed for every day. The comparisons of the parameters of body weight, daily running activity and running activity in the different periods (id est FAA, PA, NA, PRA) between ABA and control mice within each phase of starvation were evaluated by two-way ANOVA with repeated measurements with a significance level of 5%. The parameters wheel time, house time, outside time, as well as outside activity were also analyzed using two-way ANOVA with repeated measurements. Therefore, light phase I (3 p.m. to 6 p.m.), dark phase (6 p.m. to 6 a.m.) and light phase II (6 a.m. to 1 p.m.) were predefined. Bonferroni’s test was performed for post hoc comparisons between the control and ABA groups. The occurrence of the estrous phase was tested within 4 days. Chi-squared tests were used to test if the estrous phase in every 4-day block was significantly altered between ABA and control mice. All the above analyses were conducted using SPSS version 20 for Windows (IBM, Chicago, IL, USA).

## 3. Results

### 3.1. Chronic Starvation Induced AN-Related Symptoms of Hyperactivity and Amenorrhea

**Figure 3:**
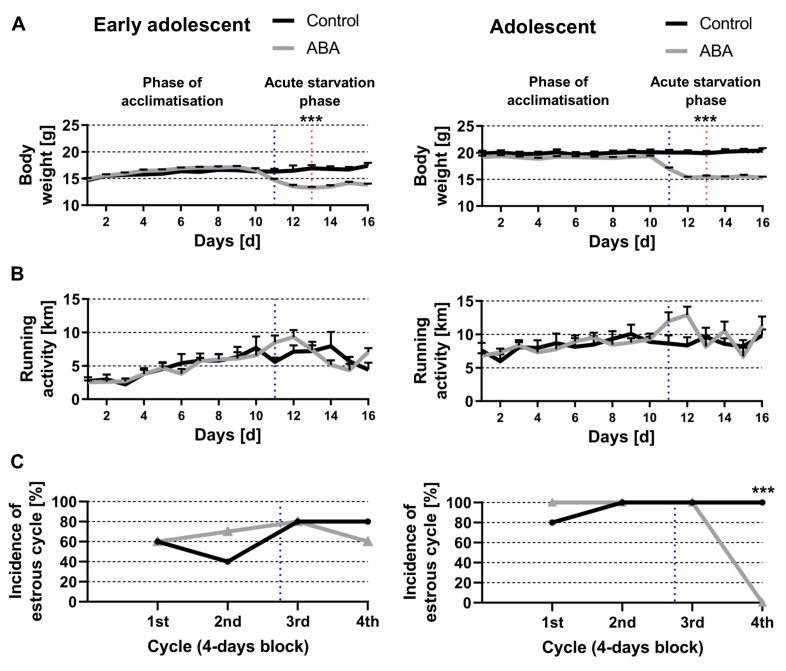
Acute starvation induces amenorrhea in adolescent mice. (**A**) The body weight, (**B**) running wheel activity measured by sensors, and (**C**) incidence of estrous cycle determined by histological examination of vaginal smears during acute starvation in early adolescent (left) and adolescent mice (right). The blue dotted lines represent the start of acute starvation phases and the red dotted line when ABA groups achieved a 20% body weight loss. The occurrence of the estrous cycle was determined in 4-day blocks based on the normal duration of the cycle in mice. (**A**,**B**) *** *p* ≤ 0.001, two-way ANOVA with repeated measurements. (**C**) Chi-squared tests comparing the incidence of the estrous cycle in ABA and control mice.

First, we determined whether acute starvation induces AN-related symptoms. The average body weight at the start of the study was 14.14 g (±0.2) for the early adolescent and 18.5 g (±0.22) for the adolescent mice. As expected, the early adolescent mice demonstrated a lower starting body weight in comparison to the adolescent mice (Figure 3A). On day 11, the start of the starvation phase (blue dotted line in Figure 3A), the ABA mice showed a 20% body weight loss within two days (i.e., on day 13 ABA_acute_4W: 13.26 g ± 0.19, ABA_acute_8W: 15.49 g ± 0.26, red dotted line in Figure 3A). Acute starvation did not alter the running activity in both ABA groups compared with the control groups (Figure 3B).

After the first 4 days block, a regular estrous cycle was detected in 60% of the control and early adolescent ABA mice indicating cycle variations probably due to the young age of the mice (Figure 3C, left). Furthermore, a regular estrous cycle was revealed in 40% of the control and 70% of the early adolescent ABA mice after the second block. At the end of the third block, a regular estrous cycle was detected in 80% of the control and early adolescent ABA mice. After the fourth block, 80% of the control and 60% of the early adolescent ABA mice showed a regular estrous cycle, indicating that acute starvation did not lead to amenorrhea.

Next, we studied adolescent animals demonstrating that a regular estrous cycle was detected in 80% of the control and all adolescent ABA mice after the first block (Figure 3C, right). After the second and third block, a regular estrous cycle was detected in all control and adolescent ABA mice. After the fourth block, a regular estrous cycle was detected in all control mice, whereas none of the adolescent ABA mice (chi-square test, χ(df) = 1, χ^2^ = 15, *p* = 0.0001). In summary, acute starvation in adolescent mice led to amenorrhea but not hyperactivity, suggesting that acute starvation is not sufficient to mimic core AN-related symptoms.

**Figure 4:**
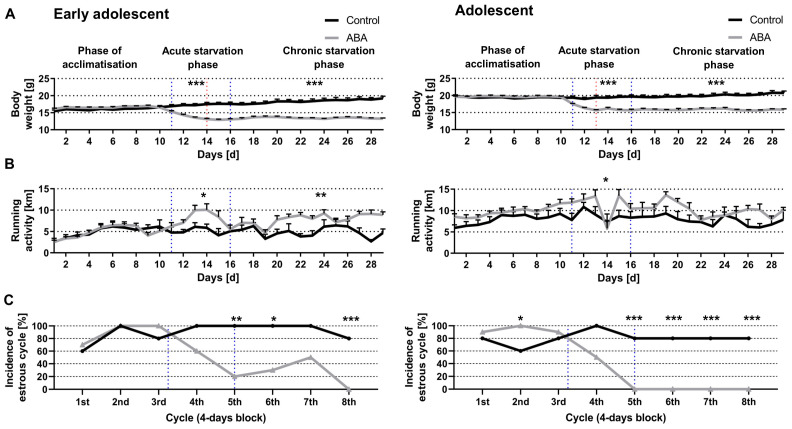
Chronic starvation in ABA mice leads to hyperactivity and amenorrhea. (**A**) The body weight, (**B**) running wheel activity explored with sensors and (**C**) incidence of the estrous cycle during chronic starvation in early adolescent (left) and adolescent mice (right). The blue dotted lines show the beginning of acute and chronic starvation phases, whereas the achievement of 20% body weight loss in ABA groups is presented with red dotted lines. (**A**,**B**) * *p* ≤ 0.05, ** *p* ≤ 0.01, *** *p* ≤ 0.001, two-way ANOVA with repeated measurements. (C) Chi-squared tests investigating the incidence of estrous cycle in control and ABA mice.

Next, we determined whether chronic starvation led to AN-related symptoms in a second cohort of mice (Figure 4). Chronic starvation comprises an acute starvation phase (days 11–16) followed by a chronic starvation phase (days 17–29). The early adolescent ABA mice reached a 20% body weight loss on day 14 whereas the adolescent ABA mice already on day 13 (ABA_chronic_4W: 13.12 g ± 0.23, ABA_ chronic _8W: 15.68 g ± 0.19; red dotted line in Figure 4A). In contrast to the first experiment (Figure 3), acute starvation induced a significant increase in running activity in both ABA groups (Control_chronic_4W: 5.15 km ± 0.15 vs. ABA_chronic_4W: 7.76 km ± 1.11, *p* = 0.01; Control_chronic_8W: 8.66 km ± 1.60 vs. ABA_chronic_8W: 11.35 km ± 1.35, *p* = 0.04). Furthermore, while chronic starvation induced an increase in running activity in early adolescent ABA mice, a trend was only shown for the adolescent ABA mice (Control_chronic_4W: 4.82 km ± 1.12 vs. ABA_chronic_4W: 7.99 km ± 0.76, *p* = 0.002; Control_chronic_8W: 7.57 km ± 1.60 vs. ABA_chronic_8W: 10 km ± 0.97, *p* = 0.08).

After the first four blocks (and after block 7), no significant difference was detected when analyzing the incidence of the estrous cycle between control and early adolescent ABA mice (Figure 4C, left). After block 5, a regular estrous cycle was observed in all controls and in only 20% of the early adolescent ABA mice (χ(df) = 1, χ^2^ = 8.57, *p* = 0.003). After the sixth block, a regular estrous cycle was observed in all control and only in 30% of the early adolescent ABA mice (χ(df) = 1, χ^2^ = 8.56, *p* = 0.01). Furthermore, after the last block, a regular estrous cycle was detected in 80% of the control and none of the ABA mice (χ(df) = 1, χ^2^ = 10.91, *p* = 0.001), indicating that chronic starvation led to amenorrhea.

These results were compared with the adolescents’ cohorts. After the first, third, and fourth blocks, no significant difference was detected in the incidence of estrous cycle between control and adolescent ABA mice (Figure 4C, right). After the second block, a regular estrous cycle was detected in 60% of controls whereas all adolescent ABA mice (χ(df) = 1, χ^2^ = 4.62, *p* = 0.03) displayed a regular estrous cycle. After the fifth block, a regular estrous cycle was detected in 80% of the control but in none of the adolescent ABA mice (χ(df) = 1, χ^2^ = 10.91, *p* = 0.001). In the next three blocks, no regular estrous cycle was detected in the adolescent ABA mice, indicating that chronic starvation induced an amenorrhea (block 6: χ(df) = 1, χ^2^ = 10.91, *p* = 0.001, block 7: χ(df) = 1, χ^2^ = 10.91, *p* = 0.001, block 8: χ(df) = 1, χ^2^ = 10.91, *p* = 0.001). In summary, chronic starvation induced the AN-related symptoms of hyperactivity and amenorrhea.

### 3.2. Acute and Chronic Starvation Induced Food-Anticipatory Activity

**Figure 5:**
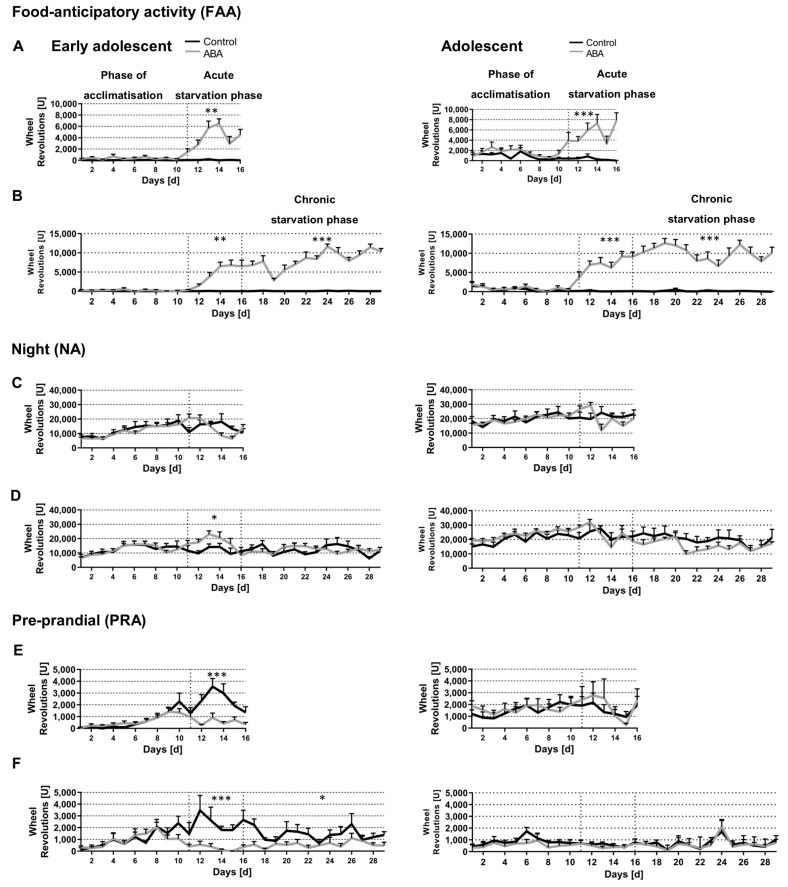
Acute and chronic starvation lead to an increase in food-anticipatory activity. (**A**,**B**) The food anticipatory activity (FAA, 4 h, from 10 a.m. to 1 p.m.), (**C**,**D**) the night activity (NA, 12 h, 6 p.m. to 6 a.m. the next day), and (**E**,**F**) the preprandial activity (PRA, 4 h, 6 a.m. to 10 a.m.) stages during acute and chronic starvation in early adolescent (left) and adolescent mice (right) were investigated using running wheel sensors. * *p* ≤ 0.05, ** *p* ≤ 0.01, *** *p* ≤ 0.001, two-way ANOVA with repeated measurements.

In order to analyze running wheel activity for different daily periods of mice, cycles of 24 h were defined into the following phases and the running activity was evaluated accordingly: FAA (4 h prior to feeding, from 9 a.m. to 1 p.m.), post-prandial activity (PA, 4 h, from 2 p.m. to 6 p.m.), night activity (NA, 12 h, 6 p.m. to 6 a.m. next day), and pre-prandial activity (PRA, 3 h, from 6 a.m. to 9 a.m., Figure 5).

During the phase of acclimatization, no differences in FAA were detectable between the different groups. Both, acute and chronic starvation induced an increase in FAA of early adolescent ABA mice in comparison to the corresponding control mice (Figure 5A,B, acute starvation phase: Control_acute_4W: 54 U ± 46 vs. ABA_acute_4W: 3980 U ± 950, *p* = 0.002; Control_chronic_4W: 14 U ± 12 vs. ABA_chronic_4W: 4579 U ± 956, *p* = 0.003; chronic starvation phase: Control_chronic_4W: 35 U ± 30 vs. ABA_chronic_4W: 8416 U ± 1009, *p* = 0.000001). Furthermore, acute and chronic starvation also led to an increased FAA in adolescent ABA mice (acute starvation phase: Control_acute_8W: 347 U ± 200 vs. ABA_acute_8W: 5346 U ± 1444, *p* = 0.001; Control_chronic_8W: 143 U ± 90 vs. ABA_chronic_8W: 7654 U ± 1356, *p* = 0.001; chronic starvation phase: Control_chronic_8W: 160 U ± 93 vs. ABA_chronic_8W: 9970 U ± 1497, *p* = 0.000001). In summary, acute and chronic starvation induced an increase in FAA in all experimental groups, further increasing during the course of starvation.

No changes in the post-prandial activity (PA) were observed in all groups (data not shown). Acute starvation led to an increase in night activity (NA) of one cohort in early adolescent (but not adolescent) ABA mice (Figure 5C,D, Control_chronic_4W: 11873 U ± 2779 vs. ABA_ chronic_4W: 16226 U ± 2774, *p* = 0.04). Interestingly, acute and chronic starvation led to a decrease in pre-prandial activity (PRA) in the early adolescent but not adolescent ABA mice (Figure 5E,F, acute starvation phase: Control_acute_4W: 2245 U ± 545 vs. ABA_acute_4W: 605 U ± 238, *p* = 0.00001, Control_chronic_4W: 2294 U ± 780 vs. ABA_chronic_4W: 335 U ± 182, *p* = 0.0002; chronic starvation phase: Control_chronic_4W: 1334 U ± 479 vs. ABA_chronic_4W: 566 U ± 291, *p* = 0.03). 

### 3.3. Starvation Decreased Circadian-Rhythm-Related Activity in Adolescent ABA Mice at Night

**Figure 6:**
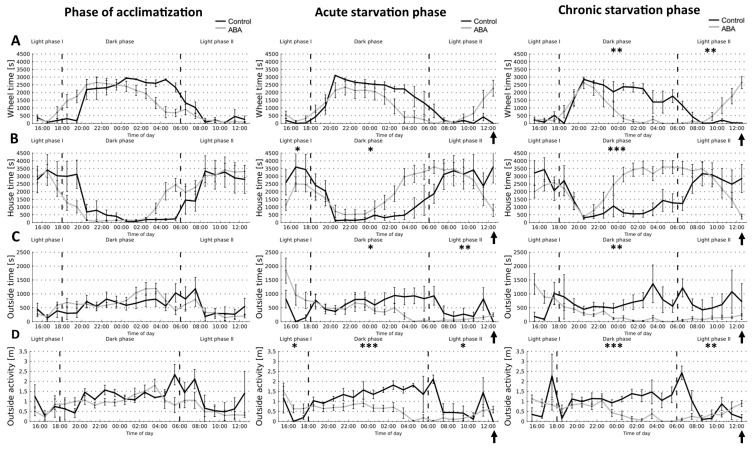
Starvation induces decreased circadian-rhythm-related activity at night. The different parameters, id est (**A**) wheel time, (**B**) house time, (**C**) outside time, and (**D**) outside activity, were measured with the tracking system Goblotrop during acclimatization, acute starvation, and chronic starvation in adolescent ABA (*n* = 8) and corresponding control mice (*n* = 4). Daily at 1 p.m., food was provided to the mice (marked with arrows). * *p* ≤ 0.05, ** *p* ≤ 0.01, *** *p* ≤ 0.001, two-way ANOVA with repeated measurements.

To determine if starvation changes the circadian-rhythm-related activity, we used the Goblotrop system for which light phase I (3 p.m. to 6 p.m.), dark phase (6 p.m. to 6 a.m.), and light phase II (6 a.m. to 1 p.m.) were predefined (Figure 6). No significant differences in all analyzed parameters were detectable between adolescent ABA and control mice during the phase of acclimatization (Figure 6A–D, left). Furthermore, acute starvation did not lead to a significant change in the wheel time of adolescent ABA mice. In contrast, chronic starvation induced a reduction in the wheel time of ABA mice in the dark phase (Control_chronic_8W: 23,125 s ± 363 vs. ABA_chronic_8W: 10,595 s ± 278, *p* = 0.008, Figure 6A). In the light phase II, during chronic starvation the wheel time of these mice steadily increased before the feeding period representing FAA behavior (Control_chronic_8W: 1875 s ± 267 vs. ABA_chronic_8W: 6167 s ± 881, *p* = 0.004).

The parameter house time reveals insights into the sleep behavior of the mice (Figure 6B). Acute starvation induced a decrease in house time in ABA mice in the light phase I (Control_ chronic_8W: 9631 s ± 3210 vs. ABA_chronic_8W: 6104 s ± 2034, *p* = 0.04). In contrast, acute and chronic starvation led to an increase in house time in ABA mice in the dark phase, indicating an increase in sleeping behavior (acute starvation phase: Control_chronic_8W: 9133 s ± 359 vs. ABA_chronic_8W: 19,344 s ± 445, *p* = 0.04; chronic starvation phase: Control_chronic_8W: 11,778 s ± 432 vs. ABA_chronic_8W: 28,948 s ± 427, *p* = 0.0001).

To assess the activity outside of the running wheel and the house, the outside time was measured (Figure 6C). During acute starvation the outside time of ABA mice was reduced in the dark phase, indicating a change in daily activity pattern (acute starvation phase: Control_chronic_8W: 8784 s ± 244 vs. ABA_chronic_8W: 4913 s ± 147, *p* = 0.02). Beyond this, the outside time of the ABA mice was decreased in the light phase II (Control_chronic_8W: 2688 s ± 384 vs. ABA_chronic_8W: 700.04 s ± 100, *p* = 0.002). Furthermore, chronic starvation led likewise to a decrease in the outside time of ABA mice in the dark phase (Control_chronic_8W: 8295 s ± 306 vs. ABA_chronic_8W: 2331 s ± 61, *p* = 0.002).

Additionally, acute starvation led to an increase in the outside activity of ABA mice in the light phase I (Control_chronic_8W: 1.35 m ± 0.45 vs. ABA_chronic_8W: 2.79 m ± 0.932, *p* = 0.03; Figure 6D). During acute starvation, the outside activity in the ABA mice was reduced in the dark phase and light phase II, paralleling the results of the outside time (dark phase: Control_chronic_8W: 16.6 m ± 0.26 vs. ABA_chronic_8W: 7.2 m ± 0.17, *p* = 0.0004; light phase II: Control_chronic_8W: 4.95 m ± 1.0 vs. ABA_chronic_8W: 1.84 m ± 0.26, *p* = 0.03). Chronic starvation led to the same effects in the dark phase and light phase II (dark phase: Control_chronic_8W: 13.0 m ± 0.27 vs. ABA_chronic_8W: 5.41 m ± 0.15, *p* = 0.001; light phase II: Control_chronic_8W: 5.1 m ± 0.73 vs. ABA_chronic_8W: 2.74 m ± 0.39, *p* = 0.004). In summary, acute and chronic starvation induced changes in circadian-rhythm-related activity in the dark and light phases.

## 4. Discussion

Hallmarks of the psychiatric disorder AN are a loss in body weight, extensive locomotor activity, and endocrinological changes [22,23]. Here, we investigated the consequence of food restriction on AN-related symptoms, namely, hyperactivity and amenorrhea, and circadian rhythms in mice. We showed that chronic starvation most closely mimics the AN-related symptoms, id est, amenorrhea, and hyperactivity. Amenorrhea predominantly occurred during chronic starvation, indicating that amenorrhea might result from a secondary hypothalamic dysfunction. The second symptom, hyperactivity, is a general enhancement in locomotor activity which can be explained by food-seeking behavior and includes frontal cerebral neuronal circuits [13]. Our study demonstrates that acute starvation did not lead to hyperactivity in all cohorts, likely due to interindividual varying adaptability to lower energy reserves, suggesting that a complex mechanism might underlie this behavior.

In addition to the general hyperactivity, the running activity at different times of the day showed that starvation increased FAA, which is regulated by hypothalamic neuronal circuits [13]. Thus, a pronounced FAA development appeared independently of starvation length and the age of the mice indicating that FAA is a direct consequence of starvation. As general hyperactivity and FAA were observable in different cohorts, two different mechanisms leading to an increase in locomotor activity might exist: firstly, a mechanism that involves food-seeking behavior, and secondly, a mechanism that involves the expectation of feeding due to a linking of feeding times to the circadian rhythm [13]. The FAA might be independent of the master clock, the SCN in the hypothalamus, since increased FAA was reported in SCN-lesioned mice and rats [24,25,26]. Thus, it was previously hypothesized that a separate food-entrainable oscillator exists [14]. Furthermore, the FAA intensity became more severe during the course of starvation indicating the need to implement experiments mimicking chronic starvation to clarify the underlying hypothalamic neuronal mechanism involved in FAA development. 

To investigate this development in detail, other diurnal activities should be observed as an activity change in FAA might lead to a shift in other daily periods. The night activity was exclusively altered in one cohort indicating that this activity is variable during starvation. In addition, pre-prandial activity was reduced in early adolescent mice only, likely reflecting an adaptation mechanism saving this energy for FAA behavior. Wu and colleagues showed that post-prandial activity in ABA mice was associated with weight loss, indicating that the FAA is not pivotal for the disease severity in the model [27]. However, no change in this activity was measured in our study. The difference in the structure of the model (1.5 h of food access per day vs. a fixed, limited amount of food per day) might be one reason for this contradiction. These results highlight that it is important to also study activities during daily periods other than FAA during starvation. 

Next, we tested the hypothesis that starvation leads to changes in circadian-related activity pattern using the Goblotrop system. We found that starvation led to a decrease in circadian-rhythm-related activity at night and an increase during the day, i.e., the outside time in ABA mice was decreased at night and the wheel time was increased during the day during chronic starvation. This is in line with the literature as an increase in activity in ABA mice during the light phase was shown previously, indicating a change in the circadian system [17,18]. Furthermore, disruptions of the circadian rhythm were also shown in patients with AN confirming this as a relevant point in the context of eating disorders [6]. The neuronal mechanism of circadian oscillation due to light is mediated by the retinohypothalamic tract, which connects the retina with the SCN (reviewed in [28]). Then, the stimulus results in a signaling cascade to the nucleus paraventricularis in the hypothalamus and finally to the pineal gland in which melatonin metabolism, an important effector of the circadian clock, is controlled. Although the C57BL/6 mice within the present study are melatonin-deficient, these mice seem to have quite similar circadian rhythms in comparison to melatonin-proficient mouse strains [29,30]. Beyond that, the circadian rhythm interacts bidirectionally with the regulation of energy balance [31], indicating that changes in the circadian rhythm might contribute to the pathophysiology of AN.

Given that the circadian rhythm and sleep–wake systems are closely associated [32], changes in circadian rhythm due to starvation might also induce disruptions in the sleep–wake system. In our study, at night ABA mice spent more time in the house and less time in the outside part of the cage, suggesting that ABA mice increased their sleep behavior during their active daytime period, id est, the dark time, indicating a disruption in sleep–wake rhythm. More detailed, the sleep pattern consists of Rapid Eye Movement (REM) and non-Rapid Eye Movement (non-REM) sleep phases which are due to different brain activities (reviewed in Ref. [33]), whereas sleep disruptions might change behavior such as aggressiveness [34]. In patients with eating disorders, changes in sleeping rhythmicity and frequent aggressiveness have been reported (reviewed in [7,35]). Furthermore, we speculate that the changes in circadian and sleep–wake rhythm patterns are associated with brain atrophy, astrocyte density reduction, and impairment of recognition memory observed in ABA rats previously shown by our group [12,36,37,38]. Interestingly, astrocytes have been identified as mediators of circadian oscillation generation in the SCN [39,40]. They thereby contribute to the circadian clock function and further contribute to the control of sleep homeostasis. This indicates that glial cell dysfunction contributes to the circadian rhythm and sleep-related activity variations during starvation. 

A limitation of this study is that the findings refer exclusively to animals. As the ABA model is the most widely used AN animal model, and the somatic consequences such as body weight loss, amenorrhea, hyperactivity, brain volume loss [36,41], and endocrinological changes (reviewed in [8,13,42,43]) demonstrate a good congruence with the symptoms of patients with AN, we assume a high translational significance. Firstly, the weight loss in the used ABA model is induced by a fixed, limited amount of food per day leading to the opportunity of chronic starvation analysis and improved interindividual comparability because of controlled weight loss. Secondly, chronic starvation in ABA mice led to amenorrhea; by comparison, this is nearly ubiquitous in AN patients. Thirdly, the hyperactivity in ABA mice can be explained by food-seeking behavior, whereas in AN patients it is commonly interpreted as a strategy to burn calories. Nonetheless, leptin treatment in ABA animals decreased hyperactivity [44], and human recombinant leptin treatment in patients with AN facilitated locomotor restlessness and depressive mood [45]. Moreover, brain atrophy was shown in a previous study in ABA rats paralleling human results [36,41]. Lastly, endocrine alterations in the ABA mice, i.e., reduced leptin and 17β-oestradiol concentrations [12], are also paralleling human findings. In summary, the ABA model appears to have a high translational capacity for patients with AN. In addition, changes in circadian rhythm and sleep-related activity which are indicated in this study were previously displayed in AN patients. Thus, it appears that these changes can be investigated with the ABA model to find new insights for the treatment of AN.

In summary, chronic starvation induced AN-related symptoms such as body weight loss, amenorrhea, and hyperactivity. Furthermore, FAA behavior seems to be a direct consequence of starvation, which might be regulated by another mechanism as general hyperactivity. Circadian-rhythm-related activity changes underlie the pathophysiology of starvation. To what extent these findings are due to neuronal dysfunction requires further analysis in both mice and humans. 

## Data Availability

The data that support the findings of this study are available from the corresponding author upon reasonable request.

## Supplementary Materials

The following supporting information can be downloaded at: https://www.mdpi.com/article/10.3390/nu14245252/s1, Figure S1: Histological determination of four stages of estrous in Giemsa-stained vaginal smears of C57BL/6J mice. Three cell types are identified: leukocytes (circle), cornified epithelial (black arrow), and nucleated epithelial (white arrow). Stages of estrous include (A) proestrous, (B) estrous, (C) metestrous, and (D) diestrous.
