# Supplementary material for: Food Restriction in Mice Induces Food-Anticipatory Activity and Circadian-Rhythm-Related Activity Changes
Source: Nutrients. 2022 Dec 9;14(24):5252. doi: 10.3390/nu14245252 (PMC9782400; doi:10.3390/nu14245252)
Supplement: Supplementary file 1 [file nutrients-14-05252-s001.zip › nutrients-2073658-supplementary.pdf]

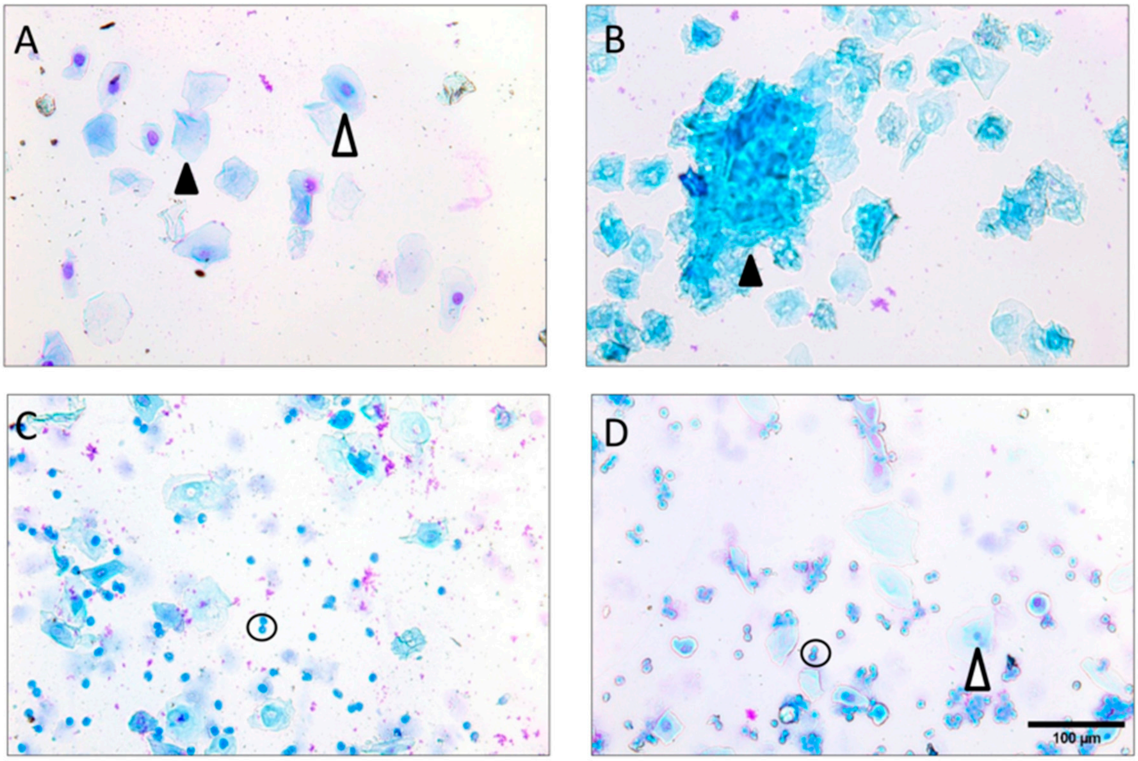

Figure S1. Histological determination of four stages of estrous in Giemsa-stained vaginal smears of C57BL/6J mice. Three cell types are identified: leukocytes (circle), cornified epithelial (black arrow), and nucleated epithelial (white arrow). Stages of estrous include (A) proestrous, (B) estrous, (C) metestrous, and (D) diestrous.
